# Apoptotic, Anti-Inflammatory Activities and Interference with the Glucocorticoid Receptor Signaling of Fractions from *Pistacia lentiscus* L. *var. chia* Leaves

**DOI:** 10.3390/plants11070934

**Published:** 2022-03-30

**Authors:** Foteini D. Kalousi, Federica Pollastro, Evgenia C. Christodoulou, Aikaterini G. Karra, Ioannis Tsialtas, Achilleas Georgantopoulos, Stefano Salamone, Anna-Maria G. Psarra

**Affiliations:** 1 Department of Biochemistry and Biotechnology, University of Thessaly, Biopolis, 41500 Larissa, Greece; fokalous@uth.gr (F.D.K.); evchristod@uth.gr (E.C.C.); aikaterini.g.karra@gmail.com (A.G.K.); tsialtasj@gmail.com (I.T.); ageorgant@uth.gr (A.G.); 2 Department of Pharmaceutical Sciences, University of Eastern Piedmont, 28100 Novara, Italy; federica.pollastro@uniupo.it (F.P.); stefano.salamone@unipo.it (S.S.); 3 PlantaChem Srls, Via Amico Canobio, 28100 Novara, Italy

**Keywords:** *Pistacia lentiscus* L., Chios, mastiha, glucocorticoid receptor, inflammation, apoptosis

## Abstract

In this study acetonic extracts of leaves of *Pistacia lentiscus* L. *var*. *chia* (mastiha tree) grown in the south as well as in the north Chios Greek island were isolated and further fractionated to give three different polarity fractions: apolar, medium-polar, and polar. The isolated fractions were assessed as regards their main composition, cytotoxic, anti-inflammatory activities, and interference with the glucocorticoid receptor (GR) signaling, applying cytotoxic assay, luciferase assays, and Western blot analysis of apoptosis-, energy-, and inflammation-associated molecules. Differences in cell viability have been detected among different polarity leaf fractions as well as among fractions of different plant origin with polar fractions showing the highest cytotoxicity. Fractions-induced anti-inflammatory activities and suppressive effects on the dexamethasone (DEX)-induced GR transcriptional activation were unveiled. The partition protocol of leaves fractions applied uncovers the enhanced glucocorticoid-associated biological activities of the medium-polar fractions, which may be associated with their enrichment in the triterpenoids that showed structural similarity with the glucocorticoids. A reduction in GR protein levels is observed by the fraction which is shown to be associated with the medium polar-induced proteolytic degradation of the receptor. In addition, the enhanced cytotoxic, anti-inflammatory, and potential anti-glycemic activities of the fractions from the Southern *P. lentiscus* L. that exclusively produce the mastiha resin, is revealed, indicating that leaves fractions from mastiha tree, similarly to mastiha tree resin, may have the potential to be further analyzed for their potent applications in the pharmaceutical cosmetic and nutraceutical fields.

## 1. Introduction

The Chios mastiha tree (*Pistacia lentiscus* L. *var. chia*) is an endemic plant cultivated on the Greek island of Chios. The aromatic resin (mastiha) exclusively secreted from the Chios mastiha tree, grown in the southern part of the island, has raised the interest of many researchers for its ethnobotanical implication in traditional medicine due to its biological properties such as anti-inflammatory, anti-bacterial, anti-oxidant, anti-cancer, cardioprotective, and hepatoprotective activities [1,2]. Recently, the European Medicines Agency, (EMA), has recognized Chios mastiha as a traditional remedy to treat mild dyspeptic disorders (functional dyspepsia), minor inflammations of the skin, and as an aid in minor wound healing [2].

The plant resin has always been the main object of studies with the scientific attention focused on the elucidation of both the biochemical mechanisms of action and the responsible secondary metabolites. Nowadays *P. lentiscus* L. leaves remain a chemical space not fully explored with the exception of essential oil of fresh and dried leaves that revealed many volatile compounds, such β-myrcene, limonene, δ-germacrene, α-cadinol, γ-cadinene, *trans*-caryophyllene, δ-cadinene from fresh vegetal material, and δ-cadinene, α-amorphene, δ-germacrene, *trans*-caryophyllene, α-cubebene, nerolidol, α-cadinene, β-cubebene, α-humulene, and naphthalene from the essential oil of dried leaves [3]. Moreover, the water extract of fresh leaves of the Chios mastiha tree was further analyzed identifying many polar compounds such as galloyl quinic acid, D-gallocathenin, myricetin-*O*-rhamnoside, quercetin glycoside, and kaempferol glycoside [4]. In addition, the chemical composition of an apolar leaf extract of *P. lentiscus* L. from Italy revealed the presence of triterpenes derivatives, such as lupeol, lupenone, β-amyrin, lupanol, and vitamin tocopherol [5].

Regarding the in vitro assessment of the biological activities of leave extracts, antioxidant activity was observed by extracts of fresh and dried leaves of the Chios mastiha tree, obtained with different extraction methods. Particularly, the highest antioxidant activity was detected in ultrasound-assisted water extracts of fresh and dried leaves [3,4]. Moreover, the essential oil obtained from leaves showed anti-bacterial activity, as indicated by the in vitro growth inhibition of Gram-positive bacteria *Staphylococcus aureus*, *Streptococcus epidermidis*, and Gram-negative bacteria *Klebsiella pneumoniae*, *Escherichia coli*. Essential oil from leaves also showed anti-fungal activity by in vitro inhibition of colonies formation of *Candida albicans* [6].

Due to triterpenoid derivatives of Italian and Chia *Pistacia lentiscus* L. leaves [3,5], which showed structural similarity with the natural glucocorticoid steroid hormone, cortisol, a crucial regulator of energy metabolism and the most highly subscribed anti-inflammatory drug, in which long-term and high doses used for pharmaceutical purposes is accompanied with many adverse side effects [7,8,9], we focused on the evaluation of the biological actions of different polarity leaves fractions as regards their interference with glucocorticoid receptor (GR) and anti-inflammatory signaling.

Thus, the study takes into consideration from the first time the acetonic extract as representative of all phytocomplex from leaves of *P. lentiscus* L. *var. chia* from trees grown both in the south and north Chios island. The acetonic extract was further sub-fractionated in different polarity fractions (apolar, medium-polar, and polar) applying the successful protocol of the European project TriForC (A pipeline for the discovery, sustainable production, and commercial utilization of known and novel high-value triterpenes with new or superior biological activities) in order to simplify the biological investigation. Then, the isolated fractions were analyzed for their biological actions in human embryonic kidney cell line HEK293 as regards their effects on cell viability, apoptosis, and interference with GR and Nuclear Factor-kappa Beta (NF-κΒ) signaling pathways. Dexamethasone (DEX), the synthetic glucocorticoid, was used as a positive control in the biological assessment assays. Comparative studies on leaves fractions from mastiha trees grown in the south and north Chios island were also performed. Finally, active fractions in the regulation of GR signaling were further analyzed through protonic nuclear magnetic resonance (^1^H NMR) spectrometry for the identification and quantification of their triterpenoid components.

## 2. Results

### 2.1. Effect of Chios Mastiha Tree Leaves Fractions on HEK293 Cell Viability

**Figure 1:**
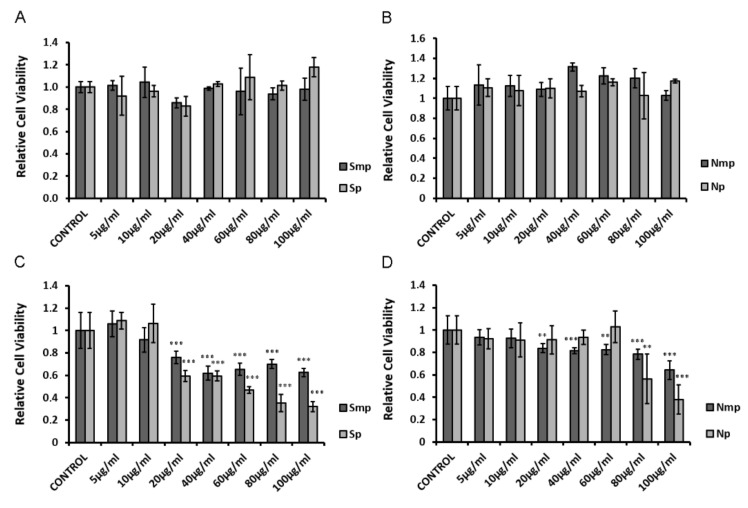
Evaluation of the effect of the Southern (**A**,**C**) and Northern (**B**,**D**) medium-polar (mp) and polar (p) leaves fractions on cell viability of HEK293 cells. Cell viability was assessed by MTT assay at two time points, 6 h (**A**,**B**) and 48 h (**C**,**D**). Relative cell viability is expressed as cell viability of the fractions at the indicated concentrations compared to control vehicle-treated cells. Cell viability of control cells was set at 1. Data are expressed as the mean ± SD, (*n* = 5–9), ** *p* < 0.01; *** *p* < 0.001.

Cell viability of medium-polar (mp) and polar (p) fractions from leaves of the *Pistacia lentiscus* L. grown in the South (S) (Figure 1A,C) and North (N) (Figure 1B,D) Chios island was evaluated by applying the MTT assay in HEK293 cells that were incubated with the indicated fractions at a range concentration of 5 μg/mL to 100 μg/mL, for 6 h (Figure 1A,B) and 48 h (Figure 1C,D). As shown in Figure 1A,B, upon 6 h incubation of the cells no statistically significant reduction in cell viability was observed by the mp and p fraction, respectively. Cytotoxic effects of the fractions were observed upon 48 h incubation (Figure 1C,D). Thus, 25–40% and 40–60% reductions in cell viability were observed by the Southern medium-polar (Smp) and Southern polar (Sp) fractions, respectively, at a concentration range of 20–100 μg/mL (Figure 1C), while both Northern medium-polar (Nmp) and Northern polar (Np) fractions are less toxic, showing a 20–60% reduction in cell viability at higher concentrations of 80–100 μg/mL (Figure 1D). Polar fractions caused an increased reduction in cell viability compared to the medium-polar ones. Apolar fractions (ap), showed no cytotoxicity at a concentration of 50 μg/mL, compared to vehicle-treated (1/1000 EtOH) cells. Due to the cytotoxic effect of EtOH, at a dilution lower than 1/1000, 50 μg/mL was the highest concentration of ap fractions applied (Supplementary data Figure S1).

### 2.2. Chios Mastiha Tree Medium-Polar and Polar Leaves Fractions Suppressed the DEX-Induced GR Transcriptional Activation

**Figure 2:**
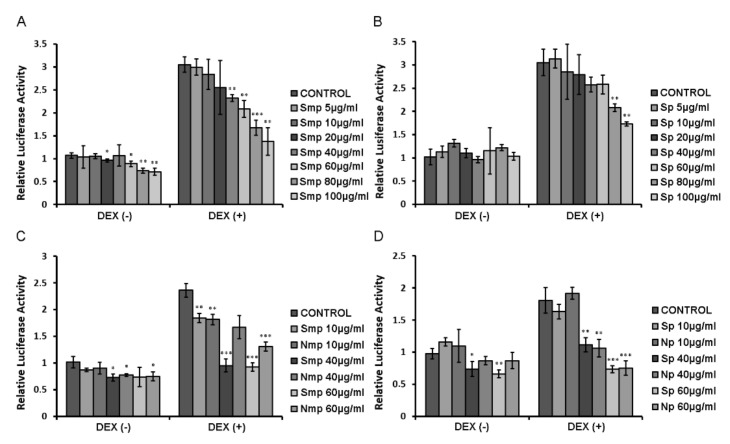
Suppression of the DEX-induced GR transcriptional activation by South (**A**,**B**) and South versus North (**C**,**D**) Chios mastiha tree leaves fractions. Luciferase and β-galactosidase activity were assessed in cell extracts from HEK293 in hormone-free medium, transiently co-transfected with a Glucocorticoid Response Elements (GRE)-Luc reporter gene construct and a β-galactosidase reporter construct and subsequently treated with 5–100 μg/mL of leaves fractions and/or 1 μΜ DEX, for 6 h. Control cells were treated with DMSO (1:1000) and EtOH (1:1000). Relative luciferase activity was expressed as luciferase activity normalized against β-galactosidase activity. Data are expressed as the mean ± SD, (*n* = 6–9), * *p* < 0.05; ** *p* < 0.01; *** *p* < 0.001, compared to relative controls.

Effect of leaves fractions on GR transcriptional activation was studied applying luciferase reporter gene assay (Figure 2). HEK293 cells grown in hormone-depleted medium were transfected as described in the experimental section and incubated with the indicated fractions, at a concentration range of 5–100 μg/mL, in the presence or absence of 1 μΜ DEX, for 6 h. Relative luciferase activities are presented in Figure 2. No GR transcriptional activation was observed by the fractions. As was expected, DEX induced two to three folds induction in GR transcriptional activation. Interestingly, a statistically significant suppression (approximately 20–50%) of the DEX-induced GR transcriptional activation was observed at a concentration range from 40 μg/mL to 100 μg/mL by the Smp fraction (Figure 2A). Similarly, a statistically significant reduction in GR transcriptional activation (up to 30–40%), compared to the DEX-treated cells, was also detected by the Sp fraction, although at higher concentrations of 80 μg/mL and 100 μg/mL (Figure 2B). Thus, we conclude that the Smp fraction is more active in inducing suppression of the DEX-induced GR transcriptional activation than the polar one. South apolar fraction (Sap) showed no statistically significant regulatory effect on the GR transcriptional activation at a concentration of 50 μg/mL, whereas the North apolar (Nap) fraction showed approximately 50% inhibition of the DEX-induced GR transcriptional activation (Supplementary data Figure S2A). In addition, comparative studies focusing on the assessment of the effect of leaves fractions of *P. lentiscus* L. grown in the South versus (vs) North Chios revealed that the Smp fraction is 20–30% more active in inducing suppression of the DEX-induced GR transcriptional activation than the North one (Figure 2C), while no statistically significant differences were observed between the polar fractions (Figure 2D).

**Figure 3:**
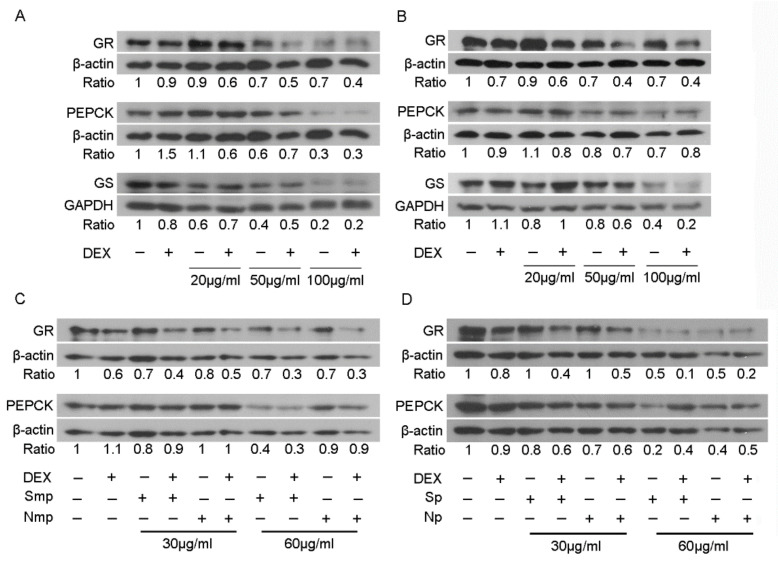
Effect of medium-polar (**A**,**C**) and polar (**B**,**D**) leaves fractions from mastiha trees grown in south (**A**,**B**) and south versus north (**C**,**D**) Chios on GR, PEPCK, and/or GS protein levels. Western blot analysis of GR, PEPCK, and GS protein levels in cell extracts from HEK293 cells treated with 20, 30, 50, 60, and 100 μg/mL of leaves fractions and/or 10 nM DEX, for 48 (**C**,**D**) and 72 h (**A**,**B**) in hormone depleted medium, was performed using commercially provided antibodies. Ratios express normalization of bands intensity compared to the respective β-actin or glyceraldehyde -3-phosphate dehydrogenase (GAPDH) ones.

The inhibitory effect of leaves fractions on the DEX-induced GR transcriptional activation was further assessed applying Western blot analysis of GR and its targets phosphoenolpyruvate carboxykinase (PEPCK) and glutamine synthetase (GS) [10,11], in extracts from HEK293 cells treated with various concentrations of mp and p fractions, varying from 20 to 100 μg/mL, in the presence or absence of 10 nM DEX or DMSO/EtOH vehicle, for 48–72 h (Figure 3). Thus, Western blot analysis of GR showed that Smp fraction (20–100 μg/mL) caused a 10–30% reduction in GR protein levels. Interestingly, co-administration of Smp fraction (20–100 μg/mL) with DEX caused an approximately 40–60% reduction in GR protein levels, compared to vehicle-treated cells (Figure 3A). Similarly, Sp fraction caused a dose-dependent reduction in GR protein levels, both in the absence (10–30% reduction) and in the presence (40–60%) of DEX, at concentrations of 20–100 μg/mL (Figure 3B). Regarding the effect of Smp and Sp fractions on the PEPCK protein level, our results showed that the Smp fraction caused a 40% and 70% reduction in PEPCK protein levels, at concentrations of 50 μg/mL and 100 μg/mL, respectively, in the presence or absence of DEX (Figure 3A). A reduction in the gluconeogenic PEPCK protein levels was also observed by the Sp fraction (50 and 100 μg/mL), although to a much lower extent of 20% and 30%, respectively, in the presence or absence of DEX (Figure 3B). Glutamine synthetase protein levels were also reduced by the fractions. Specifically, medium-polar leaves fraction of the South Chios mastiha tree (Smp) caused a 30–80% reduction in GS protein levels, at concentrations of 20–100 μg/mL, while co-administration with DEX did not cause any additive or suppressive effect (Figure 3A). Similarly, the Sp fraction caused a 20–60% reduction in GS protein levels, at concentrations of 20–100 μg/mL (Figure 3B). Co-administration with DEX caused an approximately 20% increase in the Sp-induced reduction in GS protein levels, at concentrations of 50 and 100 μg/mL.

Comparative studies of the effect of mp and p fractions from leaves of *P. lentiscus* L. grown in the south and north Chios island revealed no differential effect of Southern vs. Northern medium-polar (Figure 3C) and polar (Figure 3D) leaves fractions on GR protein levels. Interestingly, the medium-polar southern leaves fraction caused an approximately 60% reduction in PEPCK protein levels at a concentration of 60 μg/mL (Figure 3C). This effect was not observed by the respective northern one (no more than 10% reduction was observed by the Nmp fraction). Similarly, at a concentration of 60 μg/mL, Sp caused a higher reduction in the PEPCK protein level than the Np-induced one, (80% vs. 60%) (Figure 3C,D). To sum up, both mp and p fractions from *P. lentiscus* L. are active in inducing reductions in GR, PEPCK, and GS protein levels in a dose-dependent manner. A reduction in the gluconeogenic PEPCK protein levels is more pronounced by the southern fractions and especially by the medium-polar one, possible due to their potential enrichment in triterpenoids [12]. Considering that the apolar fraction showed moderate activity on GR transcriptional regulation and its chemical composition is expected to be less enriched in triterpenoids, in conjunction with technical problems associated with its limited solubility in solvents compatible with cellular biochemical analysis, apolar fractions were not further analyzed as regards their implication in the regulation of GR signaling.

### 2.3. Proteasome Inhibitor MG-132 Inhibits Leaves Fractions-Induced GR Protein Levels Reduction

To investigate the biochemical mechanism of the leaves fractions-induced reduction in GR protein levels, fractions’ effect on GR mRNA levels and the activation of GR proteolytic degradation were assessed. No statistically significant differences were observed on GR mRNA levels in cells treated with 50 μg/mL leaves fractions and/or 0.1 μM DEX, for 4 h (Supplementary data Figure S3).

**Figure 4:**
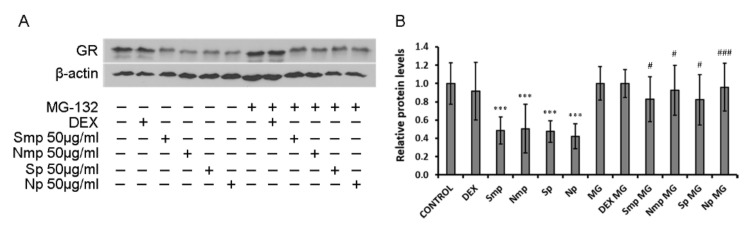
Activation of GR proteolytic degradation by leaves fractions. (**A**) Western blot analysis of GR was performed using commercially provided antibodies to evaluate GR and β-actin protein levels in HEK293 cell extracts pre-treated with 5 μM MG-132 for 1 h, and subsequently treated with vehicle or 50 μg/mL leaves fractions or 10 nΜ DEX, for 24 h in hormone depleted medium. (**B**) Quantification of the results. GR protein levels were normalized against the respective β-actin protein levels. Data are expressed as the mean ± SD, (*n* = 3), *** *p* < 0.001, compared to control vehicle-treated cells. ### *p* < 0.001, # *p* < 0.05 compared to the respective MG-132-treated cells.

Next, to assess whether a reduction in GR protein levels is attributed to possible fractions-induced proteasomal activation, HEK293 cells were subjected to pretreatment with 5 μΜ MG-132, a proteasome inhibitor [13,14], for 1 h, and then further incubated with 50 μg/mL leaves fractions or 10 nM DEX for 24 h. As shown in Figure 4, 4 h treatment with mastiha tree leaves fractions caused a reduction in GR protein levels by up to approximately 60%, as compared to control MG-132-untreated cells. Interestingly, MG-132 pre-treatment reversed the fractions-induced reduction in GR protein levels.

### 2.4. Effect of Leaves Fractions on Protein Levels of Energy Associated Molecules

A reduction in GR and its target, PEPCK, and GS, protein levels prompted us to evaluate the fractions effect on AMP-activated protein kinase (AMPK) activation. AMPK is a serine/threonine kinase which constitutes a cellular energy sensor, balancing the body’s energy levels. Thus, when intracellular ATP decreases and AMP increases, AMPK is activated by upstream kinases. Phosphorylated AMPK is activated and phosphorylates many downstream substrates, to inhibit anabolism and activate catabolism for increased energy supply and ATP production [15]. It has been shown that AMPK downstream signaling pathway interacts with GR signaling pathway, resulting in GR phosphorylation at serine 211 and the regulation of GR transactivation activity [16]. Furthermore, it has been shown that AMPK regulates the GR expression levels, and more precisely, the activation of AMPK reverses the DEX-induced reduction in GR protein levels [17]. Thus, considering that alterations in AMPK protein levels and/or its phosphorylation state could also affect GR expression and/or GR transcriptional activation, we proceeded to assess the effect of leaves fractions on AMPK expression and phosphorylation levels, applying Western blot analysis of both AMPK and p-AMPKα Thr172 (phosphorylated AMPK at threonine 172 of subunit alpha) in HEK293 cell extracts, treated with 50 μg/mL leaves fractions, for 1 h.

**Figure 5:**
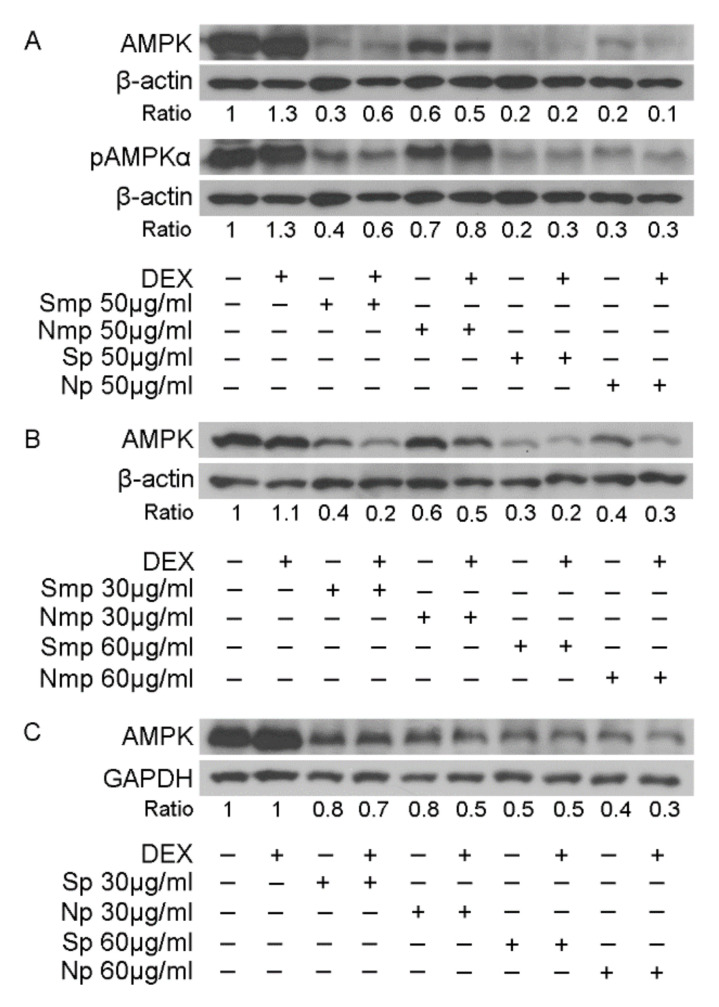
Evaluation of the effect of medium-polar and polar leaves fractions of south and north Chios mastiha tree on AMPK, pAMPKα protein levels. Western blot analysis of β-actin, GAPDH, AMPK, pAMPKα, protein levels in cell extracts from HEK293 cells treated with 30, 50, and 60 μg/mL of leaves fractions and/or 10 nM DEX, for 1 h (**A**) and 48 h (**B**,**C**) in hormone-depleted medium, was performed using commercially provided antibodies.

As shown in Figure 5A, 1 h treatment with either the medium-polar or the polar fractions caused a 40–90% decrease in protein levels of AMPK that is accompanied by a reduction in AMPKα phosphorylated form. Interestingly, the Smp fraction caused a 30% higher reduction than the Nmp fraction, while no differences between Sp and Np fractions were observed. Comparative studies on the assessment of the medium-polar to polar fractions activity revealed that polar fractions from both the southern and northern origin showed higher reduction in AMPK and phospho-AMPKα protein levels than the respective medium-polar ones, by 10% and 40%, respectively (Figure 5A). Verification of these results was also observed upon 48 h treatment of the cells (Figure 5B,C). Thus, as shown in Figure 5B,C, leaves fractions caused a dose-dependent reduction in AMPK protein levels, upon 48 h incubation, with the Smp fraction being more active than the Nmp (Figure 5B), whereas no differences between the different origin polar fractions were observed (Figure 5C). In addition, co-treatment of leaves fractions with DEX did not cause any further effect.

### 2.5. Anti-Inflammatory Actions of Chios Mastiha Tree Leaves Fractions via Suppression of the TNFα-Induced NF-κB Transcriptional Activation

**Figure 6:**
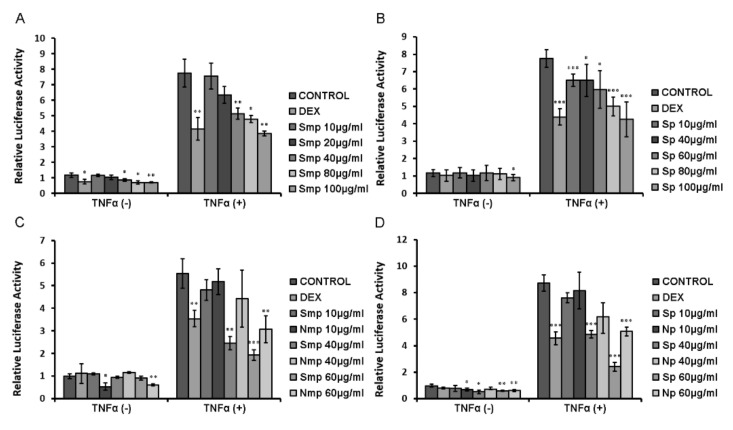
Suppression of the TNFα-induced NF-κB transcriptional activation by South (**A**,**B**) and South versus North (**C**,**D**) mp and p fractions of Chios mastiha tree leaves, in HEK293 cells. Luciferase and β-galactosidase activity was measured in cell extracts from HEK293 cells cultured in hormone-depleted medium, transiently co-transfected with an NF-κB-Luc reporter gene construct and a β-galactosidase reporter construct and subsequently treated with 5–100 μg/mL leaves fractions and/or 20 ng/mL TNFα, for 6 h. Control cells were treated with DMSO (1:1000). Relative luciferase activity was expressed as normalized luciferase activity against β-galactosidase activity. Data are expressed as the mean ± SD, (*n* = 3–6), * *p* < 0.05; ** *p* < 0.01; *** *p* < 0.001.

Potential anti-inflammatory activity of apolar (50 μg/mL), medium-polar and polar leaves fractions (10–100 μg/mL) of the Chios mastiha tree was evaluated via assessment of the inhibition of the Tumor Necrosis Factor alpha (TNFα)-induced transcriptional activation of the inflammatory factor NF-κΒ, applying a luciferase assay, using NF-κB-RE-luciferase and β-galactosidase constructs. Our results showed that south medium-polar fraction at a concentration range of 40 μg/mL to 100 μg/m caused an approximately 30–50% statistically significant inhibition of the TNFα-induced NF-κΒ transcriptional activation (Figure 6A). Similarly, the south polar fraction caused up to a 45% dose-dependent statistically significant reduction in the TNFα-induced NF-κB transcriptional activation (Figure 6B). Comparative studies on the effects of the southern/northern mp and p fractions showed that fractions from leaves of the south Chios mastiha tree exhibited higher inhibition of the TNFα-induced NF-κΒ transcriptional activation compared to the respective ones by leaves fractions from the north Chios *Pistacia lentiscus* L. (Figure 6C,D). No anti-inflammatory effect was observed by 50 μg/mL Sap, whereas approximately 35% inhibition was observed by the Nap fraction (Supplementary data Figure S2B).

**Figure 7:**
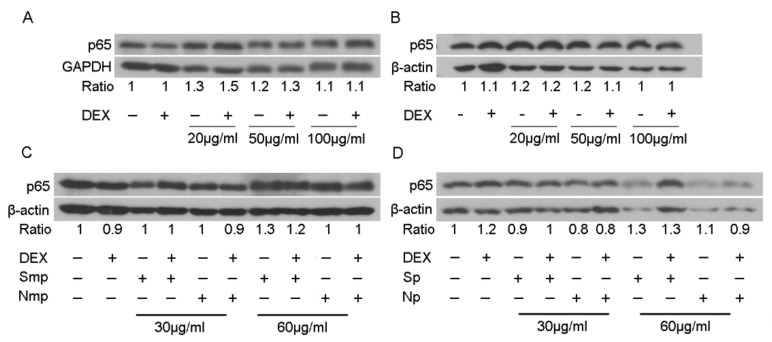
Regulation of the p65 protein levels by medium-polar (**A**,**C**) and polar (**B**,**D**) leaves fractions from south (**A**,**B**) and south versus north (**C**,**D**) Chios mastiha tree. Western blot analysis of p65 protein levels of NF-κΒ subunit in cell extracts from HEK293 cells treated with 20 to 100 μg/mL of leaves fractions and/or 10 nM DEX, for 48 (**C**,**D**) and 72 h (**A**,**B**) in hormone-depleted medium. Ratios express normalization of bands intensity compared to the respective β-actin or GAPDH ones.

To shed light on the anti-inflammatory mechanism of actions of leaves fractions from the Chios mastiha tree, an assessment of the fractions effect on the protein levels of the p65 subunit of NF-κΒ was performed, applying Western blot analysis (Figure 7). Thus, HEK293 cells were subjected to treatment with leaves fractions at various concentrations of 20 to 100 μg/mL, in the presence or absence of 10 nM DEX or DMSO/EtOH, for 48–72 h. Interestingly, no remarkable changes in the p65 protein levels expression were observed, upon treatment of the cells with the medium-polar and polar leaves fractions, indicating that the above-mentioned fractions-induced reduction in the TNFα-induced NF-κΒ transcriptional activation is attributed to the fractions effect on the NF-κΒ transcriptional regulation rather than to their suppressive effect on the NF-κΒ subunits protein expression.

### 2.6. Interference of Chios Mastiha Tree Medium-Polar and Polar Leaves Fractions with Apoptosis

**Figure 8:**
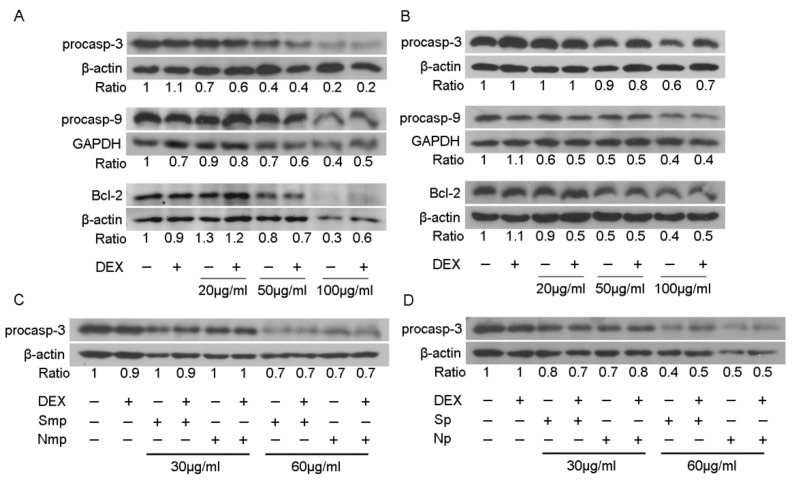
Induction of apoptosis by medium-polar (**A**,**C**) and polar (**B**,**D**) leaves fractions of South (**A**,**B**) and South versus North (**C**,**D**) Chios mastiha tree. Western blot analysis of β-actin, GAPDH, procaspase-3, procaspase-9 and Bcl-2 protein levels in cell extracts from HEK293 cells treated with 20 to 100 μg/mL of leaves fractions and/or 10 nM DEX, for 48 (**C**,**D**) and 72 h (**A**,**B**) in hormone-depleted medium was performed using commercially provided antibodies. β-actin or GAPDH protein levels were assessed for the normalization of the results.

Apoptotic activities of Chios mastiha tree leaves fractions were evaluated by applying Western blot analysis of apoptosis-associated molecules, such as procaspase-9, procaspase-3, and B-cell lymphoma-2 (Bcl-2). For that purpose, HEK293 cells were treated with 20 to 100 μg/mL leaves fractions, in the presence or absence of 10 nM DEX or DMSO/EtOH, for 48–72 h. As shown in Figure 8, the medium-polar leaves fraction of the south Chios mastiha tree caused a 30–80% reduction in procaspase-3 protein levels at a concentrations range of 20–100 μg/mL (Figure 8A). Moreover, a reduction in procaspase-9 protein levels by 30–60%, upon treatment with 50–100 μg/mL, in the absence or presence of DEX was also observed (Figure 8A). A similar pattern of reduction was also observed in Bcl-2 protein levels (Figure 8A). The polar leaves fraction from the south Chios mastiha tree caused an approximately 10–40% and 50–60% reduction in procaspase-3 protein levels and Bcl-2 protein levels, respectively, at a concentration range of 50–100 μg/mL, in the absence or presence of DEX (Figure 8B). Interestingly, the south polar fraction caused an approximately 40–60% reduction in procaspase-9 protein levels even at a low concentration of 20 μg/mL, in the absence or presence of DEX (Figure 8B). Furthermore, comparative studies of the effects of south and north medium-polar (Figure 8C) and polar (Figure 8D) leaves fractions on procaspase-3 protein levels revealed no differential effects and thus we did not further proceed to comparative studies on the apoptotic mechanisms.

### 2.7. Fractions Chemical Characterization

The fractionation protocol applied to *P. lentiscus* leaves in this work aimed to simplify the analysis, focusing on the chemical characterization of the GR-signaling most bioactive fractions. In this context, we pursued the purification looking for triterpenoids in the medium-polar fractions. The apolar and polar fractions, instead, were analyzed by ^1^H NMR for the qualitative characterization of the major compounds.

The apolar fractions of southern and northern *P. lentiscus* leaves are concentrated in unsaturated fatty acid triglycerides (Table 1), as expected from a separation with Pe as the mobile phase. These triglycerides are detectable by ^1^H-NMR from the typical presence of protons on double bounds overlapping at δ_H_ = 5.38–5.36 ppm, the allylic protons at δ_H_ = 2.80 and 2.35 ppm, the ester group at δ_H_ = 2.25 ppm as a triplet, the fatty chains protons at δ_H_ = 1.30–1.27 ppm, and the typical glycerol moiety at δ_H_ = 4.30–4.15 ppm. In these fractions, triterpenoids are marginally present (Table 1).

Both polar fractions have been purified by vacuum filtration on RP C-18 silica gel with methanol in order to avoid chlorophylls and concentrate the phenolic compounds (25.97% in southern methanolic polar fraction, 36.58% in northern methanolic polar fraction) detectable as a mixture by ^1^H-NMR due to the clear chemical shifts between δ_H_ = 7.6–6.4 ppm as doublets belonging to *p*-substituted aromatic systems and singlets at δ_H_ = 3.90–3.87 ppm revealing methoxy moieties (Supplementary data_Figure S7). The presence of triterpenoids has been identified in the residual tetrahydrofuran (THF) fractions from RP C-18 filtrations using ^1^H-NMR. Taken together, these observations could possibly justify why the polar fractions exert moderate activity on the regulation of GR signaling, showing also the highest cytotoxicity.

**Figure 9:**
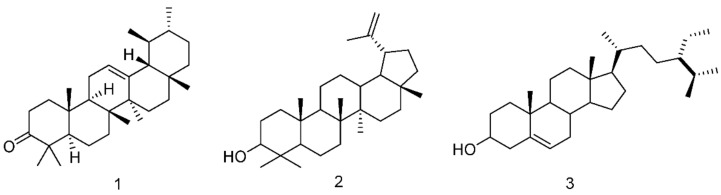
Chemical structure of α-amyrenone (**1**), lupeol (**2**), β-sitosterol (**3**).

Medium-polar fractions from *P. lentiscus* leaves extracts have been purified by chromatography, identifying triterpenoids. Not surprisingly, fractions obtained from leaves collected in the southern and northern parts of Chios island revealed differences in triterpenoids composition and quantification. The southern medium-polar fraction is characterized by 15.28% of α-amyrenone (**1**, Supplementary data Figure S4, Figure 9) and 16.54% of lupeol (**2**, Supplementary data Figure S5, Figure 9). Instead, the northern medium-polar fraction composition still presents lupeol (**2**) but in a dramatically lower yield of 0.073%. This latter fraction is lacking in α-amyrenone (**1**), but there is the presence of 0.02% β-sitosterol (**3**, Supplementary data Figure S6, Figure 9). These variations in the qualitative and quantitative composition of the different origin fractions may be associated with the observed differential biological activity of the fractions.

## 3. Discussion

Chios mastiha, the aromatic resin of the Chios mastiha tree (*Pistacia lentiscus* L. *var. chia*), is endorsed with many biological actions which have made the resin a long-standing medicinal remedy [18,19,20,21,22,23,24,25,26,27,28]. Nevertheless, limited studies have been conducted on the investigation of the biological activities of extracts of leaves of the Chios mastiha tree focusing the attention on essential oil and aqueous extracts [3,4,6].

In this study, we took into consideration, for the first time, acetonic leaves extracts from *Pistacia lentiscus* L. grown in the south and north Chios Greek island and sub-fractionated them into three fractions of different polarity: apolar fraction, ap; medium-polar fraction, mp; and polar fraction, p, to investigate their biological activities with the aim to simplify the further characterization of the active metabolites. These fractions have been investigated with respect to their anti-proliferative and anti-inflammatory actions in the human embryonic kidney cell line HEK293. Chemical characterization of the fractions revealed enrichment of medium polar fractions in triterpenoids, as was expected. Thus, the presence of **1**, **2**, **3** triterpenoids in medium-polar leaves fractions, of both origin *Pistacia lentiscus* L. *var chia* trees was revealed by ^1^H-NMR and purification. These results are in accordance with previous observations showing the terpenoid content of different origin *Pistacia lentiscus* L. leaves [3,5]. Due to triterpenoids’ structural similarity with glucocorticoids, we focused on the investigation of the possible leaves fractions interference with GR signaling pathways. GR is involved in the regulation of a plethora of cellular functions such as cell growth and metabolism, glucose homeostasis, immune responses [29], and apoptosis [30]. Thus, the possible interference of leaves fractions with GR signaling will uncover important biological actions of the fractions. In addition, due to many adverse side effects of glucocorticoids, when administered at high doses for a long time, for therapeutical purposes [9,31], interference of leaves fractions with GR signaling may bring to light new potential steroids-like natural components that will be administered alone or in combination with glucocorticoid hormones for increased steroids efficacy and reduced side effects.

Thus, for the first time, the effect of different polarity leaves fractions from the Chios mastiha tree on the synthetic glucocorticoid dexamethasone (DEX)-induced GR transcriptional activation is revealed. Interestingly, both medium-polar and polar fractions of leaves from the south Chios mastiha tree reduced the DEX-induced GR transcriptional activation in a dose-dependent manner. More interestingly, the medium-polar fraction of leaves from the southern Chios mastiha tree was revealed to be more active than the northern one, while no differential actions of the two origin polar fractions were observed. Regarding the southern apolar fraction, no effect on GR transcriptional regulation was observed, possibly due to the lower composition of steroid-like molecules, such as triterpenoids, compared to the more active medium-polar southern fraction, as revealed by ^1^H NMR analysis. According to the chemical characterization analysis and in line with previous studies on the chemical characterization of leaf extracts of various origin [12], the apolar fraction is found to be enriched in unsaturated fatty acid triglycerides; medium-polar fractions consist of triterpenoids in different concentrations, whereas polar fractions are characterized mainly by polyphenols. Thus, the increased activity of medium-polar fractions on GR signaling regulation is in accordance with the chemical composition of the fraction [5,32,33]. The observed suppressive effects of the fractions on the DEX-induced GR transcriptional activation uncover the importance of a deeper investigation to better characterize the secondary metabolites responsible for this activity. Active metabolites could constitute potential agents that when administered alone or in combination with DEX could lead to the suppression of the side effects of the GCs anti-inflammatory signaling pathway [34].

The effect of leaves fractions on the GR transcriptional activation was also studied regarding the regulation of GR target genes expression. In this context, PEPCK and glutamine synthetase protein levels were studied, upon the administration of medium-polar and polar fractions. In accordance with transcriptional activation studies, both medium-polar and polar fractions caused a reduction in PEPCK and GS protein levels. This effect is more pronounced at concentrations higher than 50 μg/mL, in the absence or presence of DEX. In comparative studies, medium-polar and polar southern leaves fractions were revealed to be more active than the northern one, in the presence or absence of DEX. The reduced PEPCK protein levels indicate potential anti-hyperglycemic actions of leaves fractions of the Chios mastiha tree, contributing also to the possible elimination of the negative side effects of GCs signaling, when co-administered with the fractions, for therapeutical purposes [31]. We propose that the increased southern medium-polar anti-gluconeogenic activity is associated with the triterpenoids’ composition of the fraction, especially lupeol (**2**) and α-amyrenone (**1**), as revealed by ^1^H-NMR analysis and the chemical characterization of the fraction. Our results are in line with previous observations demonstrating the anti-diabetic action of lupeol [35,36] and a-amyrenone [37]. Our results are also in agreement with the results of a recent study, revealing that Algerian *P. lentiscus* L. leaves extract exhibited hypoglycemic actions by reducing blood glucose levels in diabetic rats in vivo and pancreatic a-amylase activity in vitro [38]. Interestingly, we showed that GR protein levels were also affected by the leaves fractions. Particularly, co-administration of both medium-polar and polar fractions from leaves of the south Chios mastiha tree with DEX caused up to a 60% reduction in GR protein levels, while up to a 30% reduction was also observed in the absence of DEX. In comparative studies, no differential effects on GR protein levels were observed by the medium-polar and polar fractions of the two origins. Since PEPCK is a target gene of GR [10], a reduction in GR protein levels by leaves fractions could contribute to the observed reduction in PEPCK protein levels. Interestingly, we showed that reduction in GR protein levels could be attributed to fractions-induced activation of GR proteolytic degradation, as indicated by the reversal of this effect by the proteasome inhibitor, MG-132.

Moreover, in this study, we showed for the first time that leaves fractions of the Chios mastiha tree caused a reduction in AMPK protein levels, which is accompanied by a reduction in phospho-AMPKα protein levels. Polar fractions were revealed to be more active than the medium-polar ones, whereas the southern medium-polar fractions are more active than the northern ones. AMPK is involved in anabolism inhibition and catabolism activation, when AMP levels are increased, and thus an increase in energy supply is needed [15]. Interestingly, previous studies have shown that GCs treatment induced a reduction in phosphorylated AMPKα protein levels, followed by a reduction in GR protein levels, in cultured rat prefrontal cortical astrocytes [17]. Furthermore, previous studies showed that AMPK-induced activation of its downstream substrate, p38 MAPK, led to GR phosphorylation at Ser211, resulting in the regulation of GR transcriptional activation and its target genes expression, in a tissue-specific manner [16]. Thus, the fractions-induced reduction in GR transcriptional activity, GR, and its target genes PEPCK and GS protein levels, could be attributed to the mastiha tree leaves fractions-induced reduction in AMPK and its phosphorylated form.

Glucocorticoids are well-known anti-inflammatory drugs due to their transrepressional activity on NF-κΒ actions. Evaluation of the possible anti-inflammatory activity of leaves fractions in HEK293 cells showed that the medium-polar and polar, but not the apolar, fractions from the south Chios mastiha tree reduced the TNFα-induced NF-κB transcriptional activation. In comparative studies, medium-polar and polar fractions from the southern Chios mastiha tree were revealed to be approximately 1.5–2.0-fold more active than the Northern ones. This effect is attributed to the fractions effect on NF-κΒ transcriptional regulation since no effect on the regulation of the p65 subunit of NF-κB protein expression was observed. Lupeol is known for its anti-inflammatory activity [39,40]. Thus, the higher concentration of lupeol in the southern medium-polar fraction compared to the northern one, assessed by chromatography analysis, may contribute, at least in part, to the observed Smp increased anti-inflammatory activity. The anti-inflammatory activity of *Pistacia lentiscus* L. leaves fractions of other origins has also been reported. Thus, *Pistacia lentiscus* L. leaves extracts from Palestine are shown to cause a reduction in TNFα and IL-6 protein levels in lipopolysaccharide-stimulated polymorphonuclear cells [41]. Moreover, *P. lentiscus* L. leaves essential oil from Sardinia exhibited anti-inflammatory actions by inhibiting cyclooxygenases (COX-1 and COX-2) and lipoxygenase (LOX) activity, in vitro [34]. As mentioned in the introductory section, galloyl quinic acid is a major flavonoid compound that has been detected in *P. lentiscus* L. *var*. *chia* leaves [4]. Interestingly, quinic acid derivatives exhibited anti-inflammatory actions, via NF-κB inhibition to the same extent as dexamethasone [42]. Thus, anti-inflammatory activity of leaves fractions of *Pistacia lentiscus* L. *var. chia* may be attributed, at least in part, to quinic acid derivatives found to be present in *P. lentiscus* L. *var. chia* leaves extract [4] and/or to the enriched steroid-like triterpenoid composition in the medium-polar southern fraction unveiled by ^1^H-NMR analysis and characterization.

Apoptotic activities of *P. lentiscus* L. leaves extract of different origin have been previously reported in the literature. Thus, Italian *P. lentiscus* L. leaves extract was shown to inhibit human neuroblastoma SH-SY5Y and SK-N-BE(2)C cell proliferation and induced apoptotic caspase-3 activation, in vitro [5]. Anti-proliferative activities of Sardinian *P. lentiscus* L. leaves fraction, inhibiting human neuroblastoma SH-SY5Y and SK-N-BE(2)C cell proliferation and induced apoptotic caspase-3 activation, were also reported [5]. In this study, we also showed for the first time that leaves fractions of the Chios mastiha tree exhibit anti-proliferative and apoptotic actions, which are probably mitochondrial-mediated, as indicated by the leaves’ fractions-induced reduction in procaspase-9 and -3 protein levels. Medium-polar and polar leaves fractions from the south Chios mastiha tree caused an increased reduction in cell viability compared to the northern ones, while the polar fractions were revealed to be more cytotoxic than the medium-polar ones. The polar fraction from the south Chios mastiha tree seems to be more active in the mitochondrial-associated induction of apoptosis Kaempferol glycoside, a flavonoid compound, which has also been characterized as a component of the *P. lentiscus* L. *var. chia* leaves [4], which is proposed to be involved in the induction of apoptosis in human hepatocarcinoma HepG2 cells by increasing cleaved caspase-3, caspase-7, and caspase-9 protein levels [43]. In this study, a similar pattern of reduction was also observed in the case of Bcl-2 protein levels. Thus, apoptotic activities of the fractions could have potential applications in cancer treatment. Dose dependency of apoptotic actions of the fractions should also be taken into consideration in potential future applications of the fractions, focusing on their anti-inflammatory and anti-glycemic actions.

In conclusion, medium-polar and polar leaves fractions from the south and north Chios mastiha tree exhibited dose-dependent anti-proliferative, anti-inflammatory, anti-gluconeogenic, and mitochondrial-mediated apoptotic activities. The fractions-induced reduction in the DEX-induced GR transcriptional activation, which is accompanied by a reduction in GR and its target protein levels, PEPCK and glutamine synthetase, indicates that secondary metabolites from leaves, such as triterpenoids, exert possible antagonistic effects on the DEX-mediated gluconeogenic actions. A reduction in GR protein levels may be exerted via the activation of GR proteolytic degradation, through a fractions-induced reduction in AMPK protein levels. More interestingly, fractions from leaves of the mastiha tree grown in the south Chios Island were revealed to be more active than the northern ones, indicating that leaves fractions from the mastiha tree, similarly to mastiha tree resin, may have the potential to be further analyzed for their potential applications in the pharmaceutical cosmetic and food fields.

## 4. Materials and Methods

### 4.1. Chemicals

Dulbecco’s Modified Eagle’s Medium (DMEM) and fetal bovine serum (FBS) were obtained from Invitrogen. Molecular weight protein markers were purchased from Thermo Scientific. TNFα was purchased from Immuno Tools (Friesoythe, Germany). Cocktail protease inhibitors were purchased from Roche (Mannheim, Germany). Reporter lysis buffer and luciferin were purchased from Promega Corporation (Madison, WI, USA). All other solvents and chemicals including dexamethasone (DEX) and MG-132 were purchased from Sigma-Aldrich (St. Louis, MO, USA). Silica gel 60 (70–230 mesh), Celite^®^ 545 particle size 0.02–0.1 mm, CAS 68855-54-9, pH 10 (100 g/l, H_2_O, 20 °C), neutral alumina Alugram^®^, and RP C-18 silica gel used for chromatography were purchased from Macherey-Nagel (Düren, Germany). ^1^H (400 MHz) spectra were measured on Bruker 400 NMR spectrometers. Chemical shifts were referenced to the residual solvent signal (CDCl_3_: δ_H_ = 7.26). Purifications were monitored by TLC on Merck 60 F254 (0.25 mm) plates, visualized by staining with 5% H_2_SO_4_ in EtOH and heating. Chios mastiha tree leaves were kindly provided by Chios Mastic Growers Association and Mastiha Shop.

### 4.2. Plant Material Fractionation

Leaves from *Pistacia lentiscus* L. grown in the south and north Chios island were selected during the mastiha tree pruning period, at the end of October, according to suggestions by the mastiha tree growers association. Fractionation was performed in three batches of leaves. Batches of 35 g of leaves *from Pistacia lentiscus* L. grown in the south Chios island and 78 g of leaves from *Pistacia lentiscus* L. grown the north Chios, were concurrently selected (October 2018), and an extra one of 20.12 g, was selected from a mastiha tree grown in the south Chios island (October 2017). A sample of each batch was stored in phytochemical laboratories with the codes, UPO153-2018 and UPO154-2018 and UPO131-2017, respectively. Leaves were powdered and extracted with acetone (ratio acetone/plant material 10:1 vol/weight) under stirring for 12 h. The suspensions were filtered to remove the vegetal material and the acetonic fractions were evaporated to obtain a dried extract of 0.96 g (2.7% yield, southern *Pistacia lentiscus* L. leaves), 1.93 g (2.5% yield, northern *Pistacia lentiscus* L. leaves); and of 701 mg (3.48% yield, southern *Pistacia lentiscus* L. leaves), respectively, as black gums.

Dried extracts were dissolved into a minimal amount of acetone and then silica gel was added (1:3 weight/weight g) and these latter suspensions were completely evaporated. The powder obtained in this way was stratified on a layer of Celite (1:3 weight/weight g) moistened with petroleum ether (40–60) and protected on its surface by a filter paper in a sintered funnel with a side arm for vacuum connection.

Next, solvents of increasing polarity were subsequently added: petroleum ether (Pe), ethyl acetate (EtOAc), and tetrahydrofuran (THF), added in 1:30 weight/volume mL, and were sequentially passed through the filter. The three vacuum filtrates (Pe, EtOAc, THF) from each batch were collected separately and evaporated to obtain 0.186 g of apolar fraction (0.52% yield from Pe), 0.503 g of medium-polar fraction (1.4% yield from EtOAc), and 0.178 g of polar fraction (yield 0.5% yield from THF) from acetonic leaves extract from southern *Pistacia lentiscus* L. (UPO153-2018) and 0.606 g of apolar fraction (0.77% yield from Pe), 1.09 g of medium-polar fraction (1.39% yield from EtOAc), 0.215 g of polar fraction (yield 0.27% yield from THF) from acetonic leaves extract from northern *Pistacia lentiscus* L. UPO154-2018 and 0.202 g of apolar fraction (1.03% yield from Pe), 0.158 g of medium-polar fraction (0.80% yield from EtOAc), 0.129 g of polar fraction (yield 0.66% yield from THF) from acetonic leaves extract from northern *Pistacia lentiscus* L. additional batch UPO131-2017.

Medium-polar and polar fractions were diluted in DMSO at a concentration of 100 mg/mL. Whereas the apolar fraction, showing limited solubility in DMSO, was diluted in EtOH at a concentration of 50 mg/mL.

### 4.3. Chemical Characterization of Active Fractions

Apolar fractions of acetonic leaves extract from southern and northern *P. lentiscus* were characterized by ^1^H-NMR due to the minor activity proved in the regulation of GR signaling assays, revealing the presence of unsaturated fatty acid triglycerides and traces of triterpenoids.

Next, 0.36 g of the medium-polar fraction from acetonic leaves extract from southern *P. lentiscus* was fractionated by chromatography on silica gel (18 g, Pe-EtOAc gradient from 80:20 to 50:50) to afford a mixture of triterpenoids further purified by chromatography on neutral alumina (8 mL, Pe-EtOAc gradient from 100 to 90:10) obtaining 55 mg of β-amyrenone and 59.56 mg of lupeol after solvent evaporation. The compounds were identified according to the literature [44,45].

One gram of the medium-polar fraction from acetonic leaves extract from northern *P. lentiscus* was fractionated by chromatography on silica gel (50 g, Pe-EtOAc gradient from 80:20 to 50:50) to afford a mixture of triterpenoids further purified by chromatography on neutral alumina (12 mL, Pe-EtOAc gradient from 100 to 90:10) obtaining 20 mg of β-sitosterol and 73.8 mg of lupeol after solvent evaporation. The compounds were identified according to the literature [44,46].

The polar fractions of acetonic leaves extract from southern (77 mg) and northern (123 mg) *P. lentiscus* were purified by vacuum filtration on RP C-18 silica gel (1:5 weight/weight g) with methanol and THF (1:5 weight/volume mL) affording two fractions that were further analyzed by ^1^H-NMR after solvent evaporation. Both southern and northern methanolic fractions (20 mg from southern polar fraction and 45 mg from northern polar fraction) revealed the presence of phenolic compounds, the THF fractions (15 mg from the southern polar fraction and 49 from the northern polar fraction) instead revealed residual traces of lupeol.

### 4.4. Antibodies

Monoclonal antibodies against human GR and GAPDH or polyclonal antibodies against the p65 subunit of NF-κB and PEPCK were commercially provided by Santa Cruz Biotechnology. Rabbit polyclonal antibodies against procaspase-3, Bcl-2, AMPK, and phosphorylated AMPK at threonine 172 of the subunit alpha (pAMPKα) were also commercially provided by Cell Signaling Technology, Leiden, The Netherlands. Monoclonal antibodies against β-actin (Sigma-Aldrich, St. Louis, MO, USA), glutamine synthetase (GS) (Chemicon, Temecula, CA, USA), and procaspase-9 (Cell Signaling Technology, Danvers, MA, USA) were also used.

### 4.5. Cell Culture

The human embryonic kidney HEK293 cells, characterized by high efficiency in transfections experiments, were obtained from the American Type Culture Collection (ATTC) and were maintained in DMEM, supplemented with 10% FBS, 2 mM L-glutamine, and 100 units/mL penicillin/streptomycin at 37 °C and 5% CO_2_ humidity. Then, 48–72 h before treatment, cells were cultured in phenol red-free DMEM medium supplemented with 10% charcoal-dextran-stripped FBS (charcoal-stripped FBS, CSF), 2 mM glutamine, and 100 units/mL penicillin/streptomycin.

### 4.6. Cell Viability Assay

MTT assay was applied as previously described [47]. Briefly, HEK293 cells were plated in a 96-well plate, at a density of 1.5 × 10^4^ cells/well, for 24 h in DMEM medium (4.5 g/L glucose), supplemented with 10% FBS, 2 mM L-glutamine, and 100 units/mL pen/strep. The next day, cells were treated with leaves fractions, at a wide range of concentrations (5–100 μg/mL), diluted in DMSO, and incubated for 6 and 48 h. Then, the MTT reagent was added at a final concentration of 0.5 mg/mL for 3–4 h. Finally, formazan crystals were diluted with 100% isopropanol, and absorbance was measured at 570 nm using a multimode plate reader (EnSpire, PerkinElmer, Beaconsfield, UK). Background absorbance was also measured at 690 nm, as a reference.

### 4.7. GR and NF-κB Transactivation Measurement

NF-κB and GR transcriptional activity was measured by applying the luciferase reporter gene assay as previously described [7]. Briefly, HEK293 cells grown on 24-well plates were co-transfected, using calcium phosphate, with an NF-κB-RE (NF-κΒ response elements for the assessment of NF-κB activity) or an MMTV-GRE (glucocorticoid response elements for the assessment of GR activity) promoter-driven luciferase construct (NF-κΒ-RE-luc and GRE-luc, respectively) and a β-galactosidase reporter construct, for the normalization of the results. Upon 14–16 h of transfection, cells were washed in fresh medium and the next day were triggered either by 20 ng/mL TNFα (tumor necrosis factor α) for the assessment of NF-κB activity or by 1 μM DEX for the assessment of GR activity, in the presence or absence of the indicated amounts of leaves’ fractions of the Chios mastiha tree, for 6 h. Then, cells were lysed in reporter lysis buffer and the enzymatic activities of the expressed luciferase and β-galactosidase were measured. The light emission was measured using a chemiluminometer (LB 9508, Berthold Technologies GmbH & Co.KG, Baden Wurttemberg, Germany). Relative luciferase activity was expressed as normalized luciferase activity against β-galactosidase activity (RLU).

### 4.8. Electrophoresis and Western Blotting

Cells were grown in 6-well plates for 48 h in hormone-depleted medium and incubated for an additional 1 or 48–72 h with 20–100 μg/mL of Chios mastiha tree polar and medium-polar leaves fractions and/or 10 nM DEX, as indicated. Cells were washed in PBS 1X, lysed in buffer A (20 mM Tris pH:7.5, 250 mM NaCl, 0.5% Triton, 3 mM EDTA) supplemented with cocktail protease inhibitors, DTT and PMSF. After Bradford protein determination, cell extracts were electrophoresed in discontinuous SDS-PAGE and Western blotting with specific antibodies as previously described [48]. Enhanced chemiluminescence was used for the detection of the protein bands. β-actin and GAPDH expression levels were evaluated for the normalization of the GR, PEPCK, GS, procaspase-3, procaspase-9, Bcl-2, AMPK, and phosphο-AMPKα (Thr172) expression levels. In the case of MG-132 treatment, an inhibitor of the proteasome, HEK293 cells were pre-treated with 5μΜ MG-132, or DMSO, for 1 h [13,14]. Then, the cell culture medium was replaced and HEK293 cells were further treated with 50 μg/mL leaves fractions or 10 nM DEX or DMSO, for 24 h. Cells were collected, lysed, and subjected to electrophoresis and Western blot analysis.

### 4.9. Statistical Analysis

All results are expressed as the mean ± SD. Data were analyzed by independent *t*-test or by one-way analysis of variance (ANOVA) (Figure 1 and Figure 4) or two-way ANOVA (Figure 2 and Figure 6) followed by Tukey’s post hoc test using SPSS or Stat Plus software, respectively. Differences were considered significant at a two-tailed *p*-value < 0.05.

## Figures and Tables

**Table 1:** Chemical characterization of fractions from northern and southern *P. lentiscus* leaves extract. Apolar fractions have been analyzed by ^1^H-NMR, medium-polar fractions have been characterized by chromatography purification and polar fractions have been divided into MeOH and THF fractions through RP C-18 and subsequently analyzed by ^1^H NMR.

	Apolar Fraction Composition	Medium-Polar Fraction Composition	Polar Fraction Composition
*P. lentiscus* leaves extracts from northern Chios island	Fatty acid triglycerides	Lupeol 0.073%	Phenolic compounds 36.58%
	traces of triterpenoids	β-sitosterol 0.02%	traces of triterpenoids
*P. lentiscus* leaves extracts from southern Chios island	Fatty acid triglycerides	Lupeol 16.54%	Phenolic compounds 25.97%
	traces of triterpenoids	α-amyrenone 15.28%	traces of triterpenoids

## Data Availability

All data, tables and figures are original. Details on data analysis are available from the corresponding author upon reasonable request.

## Supplementary Materials

The following supporting information can be downloaded at: https://www.mdpi.com/article/10.3390/plants11070934/s1, Figure S1: Evaluation of the effect of apolar (ap) fractions on cell viability of HEK293 cells; Figure S2: Assessment of the apolar fractions effect on (a) the DEX-induced GR transcriptional activation and (b) the TNFα-induced NF-κΒ transcriptional activation by the Southern and Northern fraction, at a concentration of 50 μg/mL; Figure S3: Real-time PCR was applied to evaluate mRNA levels of GR, in HEK293 cells; Figure S4: 1H NMR of α-amyrenone in CDCl3; Figure S5: 1H NMR of lupeol in CDCl3; Figure S6: 1H NMR of β-sitosterol in CDCl3; Figure S7: 1H NMR of polar fraction in acetoned6 from *P. lentiscus* leaves extract purified by RP C-18; Table S1: Primers used for real-time PCR measurement. Reference [49] is cited in the supplementary materials.

## References

[1] Papada E., Gioxari A., Amerikanou C., Forbes A., Tzavara C., Smyrnioudis I., Kaliora A.C. (2019). Regulation of faecal biomarkers in inflammatory bowel disease patients treated with oral mastiha (Pistacia lentiscus) supplement: A double-blind and placebo-controlled randomised trial. Phytother. Res. PTR.

[2] Pachi V.K., Mikropoulou E.V., Gkiouvetidis P., Siafakas K., Argyropoulou A., Angelis A., Mitakou S., Halabalaki M. (2020). Traditional uses, phytochemistry and pharmacology of Chios mastic gum (Pistacia lentiscus var. Chia, Anacardiaceae): A review. J. Ethnopharmacol..

[3] Bampouli A., Kyriakopoulou K., Papaefstathiou G., Louli V., Krokida M., Magoulas K. (2014). Comparison of different extraction methods of Pistacia lentiscus var. chia leaves: Yield, antioxidant activity and essential oil chemical composition. J. Appl. Res. Med. Aromat. Plants.

[4] Bampouli A., Kyriakopoulou K., Papaefstathiou G., Louli V., Aligiannis N., Magoulas K., Krokida M. (2015). Evaluation of total antioxidant potential of Pistacia lentiscus var. chia leaves extracts using UHPLC–HRMS. J. Food Eng..

[5] Piccolella S., Nocera P., Carillo P., Woodrow P., Greco V., Manti L., Fiorentino A., Pacifico S. (2016). An apolar *Pistacia lentiscus* L. leaf extract: GC-MS metabolic profiling and evaluation of cytotoxicity and apoptosis inducing effects on SH-SY5Y and SK-N-BE(2)C cell lines. Food Chem. Toxicol..

[6] Magiatis P., Melliou E., Skaltsounis A.L., Chinou I.B., Mitaku S. (1999). Chemical composition and antimicrobial activity of the essential oils of Pistacia lentiscus var. chia. Planta Med..

[7] Georgatza D., Gorgogietas V.A., Kylindri P., Charalambous M.C., Papadopoulou K.K., Hayes J.M., Psarra A.G. (2016). The triterpene echinocystic acid and its 3-O-glucoside derivative are revealed as potent and selective glucocorticoid receptor agonists. Int. J. Biochem. Cell Biol..

[8] Karra A.G., Tziortziou M., Kylindri P., Georgatza D., Gorgogietas V.A., Makiou A., Krokida A., Tsialtas I., Kalousi F.D., Papadopoulos G.E. (2020). Boswellic acids and their derivatives as potent regulators of glucocorticoid receptor actions. Arch. Biochem. Biophys..

[9] Baschant U., Culemann S., Tuckermann J. (2013). Molecular determinants of glucocorticoid actions in inflammatory joint diseases. Mol. Cell. Endocrinol..

[10] Friedman J.E., Yun J.S., Patel Y.M., McGrane M.M., Hanson R.W. (1993). Glucocorticoids regulate the induction of phosphoenolpyruvate carboxykinase (GTP) gene transcription during diabetes. J. Biol. Chem..

[11] Olkku A., Bodine P.V., Linnala-Kankkunen A., Mahonen A. (2004). Glucocorticoids induce glutamine synthetase expression in human osteoblastic cells: A novel observation in bone. Bone.

[12] Chianese G., Golin-Pacheco S.D., Taglialatela-Scafati O., Collado J.A., Munoz E., Appendino G., Pollastro F. (2019). Bioactive triterpenoids from the caffeine-rich plants guayusa and mate. Food Res. Int..

[13] Wallace A.D., Cao Y., Chandramouleeswaran S., Cidlowski J.A. (2010). Lysine 419 targets human glucocorticoid receptor for proteasomal degradation. Steroids.

[14] Chui A.J., Okondo M.C., Rao S.D., Gai K., Griswold A.R., Johnson D.C., Ball D.P., Taabazuing C.Y., Orth E.L., Vittimberga B.A. (2019). N-terminal degradation activates the NLRP1B inflammasome. Science.

[15] Mihaylova M.M., Shaw R.J. (2011). The AMPK signalling pathway coordinates cell growth, autophagy and metabolism. Nat. Cell Biol..

[16] Nader N., Ng S.S., Lambrou G.I., Pervanidou P., Wang Y., Chrousos G.P., Kino T. (2010). AMPK regulates metabolic actions of glucocorticoids by phosphorylating the glucocorticoid receptor through p38 MAPK. Mol. Endocrinol..

[17] Yuan S.Y., Liu J., Zhou J., Lu W., Zhou H.Y., Long L.H., Hu Z.L., Ni L., Wang Y., Chen J.G. (2016). AMPK Mediates Glucocorticoids Stress-Induced Downregulation of the Glucocorticoid Receptor in Cultured Rat Prefrontal Cortical Astrocytes. PLoS ONE.

[18] Paraschos S., Magiatis P., Mitakou S., Petraki K., Kalliaropoulos A., Maragkoudakis P., Mentis A., Sgouras D., Skaltsounis A.L. (2007). In vitro and in vivo activities of Chios mastic gum extracts and constituents against Helicobacter pylori. Antimicrob. Agents Chemother..

[19] Kottakis F., Kouzi-Koliakou K., Pendas S., Kountouras J., Choli-Papadopoulou T. (2009). Effects of mastic gum Pistacia lentiscus var. Chia on innate cellular immune effectors. Eur. J. Gastroenterol. Hepatol..

[20] Kaliora A.C., Stathopoulou M.G., Triantafillidis J.K., Dedoussis G.V., Andrikopoulos N.K. (2007). Chios mastic treatment of patients with active Crohn’s disease. World J. Gastroenterol..

[21] Gioxari A., Kaliora A.C., Papalois A., Agrogiannis G., Triantafillidis J.K., Andrikopoulos N.K. (2011). Pistacia lentiscus resin regulates intestinal damage and inflammation in trinitrobenzene sulfonic acid-induced colitis. J. Med. Food.

[22] Andrikopoulos N.K., Kaliora A.C., Assimopoulou A.N., Papapeorgiou V.P. (2003). Biological activity of some naturally occurring resins, gums and pigments against in vitro LDL oxidation. Phytother. Res. PTR.

[23] Dedoussis G.V., Kaliora A.C., Psarras S., Chiou A., Mylona A., Papadopoulos N.G., Andrikopoulos N.K. (2004). Antiatherogenic effect of Pistacia lentiscus via GSH restoration and downregulation of CD36 mRNA expression. Atherosclerosis.

[24] Loutrari H., Magkouta S., Pyriochou A., Koika V., Kolisis F.N., Papapetropoulos A., Roussos C. (2006). Mastic oil from Pistacia lentiscus var. chia inhibits growth and survival of human K562 leukemia cells and attenuates angiogenesis. Nutr. Cancer.

[25] Balan K.V., Prince J., Han Z., Dimas K., Cladaras M., Wyche J.H., Sitaras N.M., Pantazis P. (2007). Antiproliferative activity and induction of apoptosis in human colon cancer cells treated in vitro with constituents of a product derived from Pistacia lentiscus L. var. chia. Phytomedicine Int. J. Phytother. Phytopharm..

[26] Georgiadis I., Karatzas T., Korou L.M., Agrogiannis G., Vlachos I.S., Pantopoulou A., Tzanetakou I.P., Katsilambros N., Perrea D.N. (2014). Evaluation of Chios mastic gum on lipid and glucose metabolism in diabetic mice. J. Med. Food.

[27] Kartalis A., Didagelos M., Georgiadis I., Benetos G., Smyrnioudis N., Marmaras H., Voutas P., Zotika C., Garoufalis S., Andrikopoulos G. (2016). Effects of Chios mastic gum on cholesterol and glucose levels of healthy volunteers: A prospective, randomized, placebo-controlled, pilot study (CHIOS-MASTIHA). Eur. J. Prev. Cardiol..

[28] Andreadou I., Mitakou S., Paraschos S., Efentakis P., Magiatis P., Kaklamanis L., Halabalaki M., Skaltsounis L., Iliodromitis E.K. (2016). “Pistacia lentiscus L.” reduces the infarct size in normal fed anesthetized rabbits and possess antiatheromatic and hypolipidemic activity in cholesterol fed rabbits. Phytomedicine Int. J. Phytother. Phytopharm..

[29] Kadmiel M., Cidlowski J.A. (2013). Glucocorticoid receptor signaling in health and disease. Trends Pharmacol. Sci..

[30] Gruver-Yates A.L., Cidlowski J.A. (2013). Tissue-specific actions of glucocorticoids on apoptosis: A double-edged sword. Cells.

[31] Oray M., Abu Samra K., Ebrahimiadib N., Meese H., Foster C.S. (2016). Long-term side effects of glucocorticoids. Expert Opin. Drug Saf..

[32] Xue H., Jiang Y., Zhao H., Kollner T.G., Chen S., Chen F., Chen F. (2019). Characterization of Composition and Antifungal Properties of Leaf Secondary Metabolites from Thirteen Cultivars of Chrysanthemum morifolium Ramat. Molecules.

[33] Milia E., Bullitta S.M., Mastandrea G., Szotakova B., Schoubben A., Langhansova L., Quartu M., Bortone A., Eick S. (2021). Leaves and Fruits Preparations of Pistacia lentiscus L.: A Review on the Ethnopharmacological Uses and Implications in Inflammation and Infection. Antibiotics.

[34] Sundahl N., Bridelance J., Libert C., De Bosscher K., Beck I.M. (2015). Selective glucocorticoid receptor modulation: New directions with non-steroidal scaffolds. Pharmacol. Ther..

[35] Gupta R., Sharma A.K., Sharma M.C., Dobhal M.P., Gupta R.S. (2012). Evaluation of antidiabetic and antioxidant potential of lupeol in experimental hyperglycaemia. Nat. Prod. Res..

[36] Malik A., Jamil U., Butt T.T., Waquar S., Gan S.H., Shafique H., Jafar T.H. (2019). In silico and in vitro studies of lupeol and iso-orientin as potential antidiabetic agents in a rat model. Drug Des. Dev. Ther..

[37] Ferreira R.G., Silva Junior W.F., Veiga Junior V.F., Lima A.A., Lima E.S. (2017). Physicochemical Characterization and Biological Activities of the Triterpenic Mixture alpha, beta-Amyrenone. Molecules.

[38] Mehenni C., Atmani-Kilani D., Dumarcay S., Perrin D., Gerardin P., Atmani D. (2016). Hepatoprotective and antidiabetic effects of Pistacia lentiscus leaf and fruit extracts. J. Food Drug Anal..

[39] Liu K., Zhang X., Xie L., Deng M., Chen H., Song J., Long J., Li X., Luo J. (2021). Lupeol and its derivatives as anticancer and anti-inflammatory agents: Molecular mechanisms and therapeutic efficacy. Pharm. Res..

[40] Saleem M. (2009). Lupeol, a novel anti-inflammatory and anti-cancer dietary triterpene. Cancer Lett..

[41] Qabaha K., Ras S.A., Abbadi J., Al-Rimawi F. (2016). Anti-Inflammatory Activity of Eucalyptus Spp. And Pistascia Lentiscus Leaf Extracts. Afr. J. Tradit. Complementary Altern. Med..

[42] Zeng K., Thompson K.E., Yates C.R., Miller D.D. (2009). Synthesis and biological evaluation of quinic acid derivatives as anti-inflammatory agents. Bioorg Med. Chem. Lett..

[43] Wang J., Fang X., Ge L., Cao F., Zhao L., Wang Z., Xiao W. (2018). Antitumor, antioxidant and anti-inflammatory activities of kaempferol and its corresponding glycosides and the enzymatic preparation of kaempferol. PLoS ONE.

[44] Fotie J., Bohle D.S., Leimanis M.L., Georges E., Rukunga G., Nkengfack A.E. (2006). Lupeol long-chain fatty acid esters with antimalarial activity from Holarrhena floribunda. J. Nat. Prod..

[45] Carvalho M.G., Velandia J.R., Oliviera L.F., Bezerra F.B. (1998). Triterpenos isolados de Eschweilera longipes miers (Lecythidaceae). Química Nova.

[46] Cayme J.C., Ragasa C.Y. (2004). Structure elucidation of β-stigmasterol and β-sitosterol from Sesbania grandiflora [Linn] Pers. and β-carotene from Heliotropium indicum Linn. by NMR spectroscopy. Kimika.

[47] Mosmann T. (1983). Rapid colorimetric assay for cellular growth and survival: Application to proliferation and cytotoxicity assays. J. Immunol. Methods.

[48] Tsialtas I., Gorgogietas V.A., Michalopoulou M., Komninou A., Liakou E., Georgantopoulos A., Kalousi F.D., Karra A.G., Protopapa E., Psarra A.G. (2020). Neurotoxic effects of aluminum are associated with its interference with estrogen receptors signaling. Neurotoxicology.

[49] Gorgogietas V.A., Tsialtas I., Sotiriou N., Laschou V.C., Karra A.G., Leonidas D.D., Chrousos G.P., Protopapa E., Psarra A.G. (2018). Potential interference of aluminum chlorohydrate with estrogen receptor signaling in breast cancer cells. J. Mol. Biochem..

