# Supplementary material for: Apoptotic, Anti-Inflammatory Activities and Interference with the Glucocorticoid Receptor Signaling of Fractions from Pistacia lentiscus L. var. chia Leaves
Source: Plants (Basel). 2022 Mar 30;11(7):934. doi: 10.3390/plants11070934 (PMC9002849; doi:10.3390/plants11070934)
Supplement: Supplementary file 1 [file plants-11-00934-s001.zip › plants-1659696-supplementary.pdf]

Apoptotic, Anti-Inflammatory Activities and Interference with the Glucocorticoid Receptor Signaling of Fractions from *Pistacia lentiscus* L. *var. chia* Leaves

Foteini D. Kalousi [1], Federica Pollastro [2,3], Evgenia C. Christodoulou [1], Aikaterini Karra [1], Ioannis Tsialtas [1], Achilleas Georgantopoulos [1], Stefano Salamone [2], Anna-Maria G. Psarra [1,*]

[1] Department of Biochemistry and Biotechnology, University of Thessaly, Biopolis, 41500 Larissa, Greece
[2] Department of Pharmaceutical Sciences, University of Eastern Piedmont, Novara, Italy; e-mail@e-mail.com
[3] PlantaChem Srls, via Amico Canobio, Novara, Italy
* Correspondence: ampsarra@bio.uth.gr; Tel.: +30-2410-565221

1.1. Cell viability assay

MTT assay was applied as previously described [1]. Briefly, HEK293 cells were plated in 96-well plate, at a density of 1,5x104 cells/well, for 24 hours in DMEM medium (4,5g/L glucose), supplemented with 10% FBS, 2mM L-glutamine and 100units/ml Pen/Strep. Next day, cells were treated with Southern and Northern apolar leaves fractions, at a concentration of 50μg/ml, diluted in EtOH and incubated for 48 hours. Then, MTT reagent was added at a final concentration of 0,5mg/ml, for 3-4 hours. Finally, formazan crystals were diluted with 100% isopropanol and absorbance was measured at 570nm, using a multimode plate reader (EnSpire, Perkin Elmer, UK). Background absorbance was also measured at 690nm, as reference.

As shown in Figure S1, upon 48 hours Southern and Northern apolar fractions exhibited no statistically significant reduction in cell viability, compared to control EtOH (1/1000) treated cells.

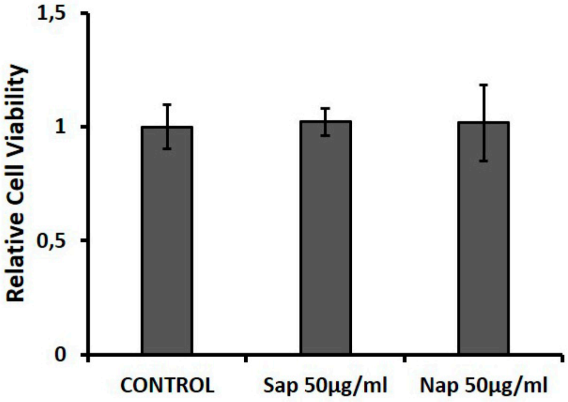

Figure S1: Evaluation of the effect of apolar (ap) fractions on cell viability of HEK293 cells. Cell viability was assessed by MTT assay at 48 hours. Relative cell viability is expressed as cell viability of the extracts at the indicated concentrations compared to control EtOH-treated cells. Cell viability of control cells was set at 1. Data is expressed as mean ± SD, (*n*=3).

1.2. GR and NF-κB transactivation measurement

NF-κB and GR transcriptional activity was measured applying luciferase reporter gene assay as previously described [2]. Briefly, HEK293 cells grown on 24-well plates were co-transfected, using calcium phosphate, with a NF-κB-RE (NF-κB response elements for assessment of NF-κB activity), or an MMTV-GRE (Glucocorticoid response elements for assessment of GR activity) promoter-driven luciferase construct (NF-κB-RE-luc and GRE-luc, respectively) and a β-galactosidase reporter construct, for the normalization of the results. 14-16 hours upon transfection cells were washed in fresh medium and the next day cells were triggered either by 20ng/ml TNFα (tumor necrosis factor α) for assessment of NF-κB activity, or by 1μM DEX for assessment of GR activity, in the presence or absence of 50μg/ml apolar fraction from leaves of the *Pistacia Lentiscus* L. of the South or North Chios Greek island, for 6 hours. Then cells were lysed in reporter lysis buffer and the enzymatic activities of the expressed luciferase and β-galactosidase were measured. The light emission was measured using a chemiluminometer (LB 9508, www.berthhold.com). Relative luciferase activity was expressed as normalized luciferase activity against β-galactosidase activity (RLU).

As shown in Figure S2A, 50μg/ml of Northern apolar fraction caused 50% statistically significant reduction in the DEX-induced GR transcriptional activation, while no changes were observed in the absence of DEX. No statistically significant differences were observed by the Southern apolar fraction. Interestingly, up to 35% suppression of the TNFα-induced NF-κB transcriptional activity were noticed upon 50μg/ml of Northern apolar fraction, while no statistically significant differences were observed by the Southern apolar fraction (Figure S2B).

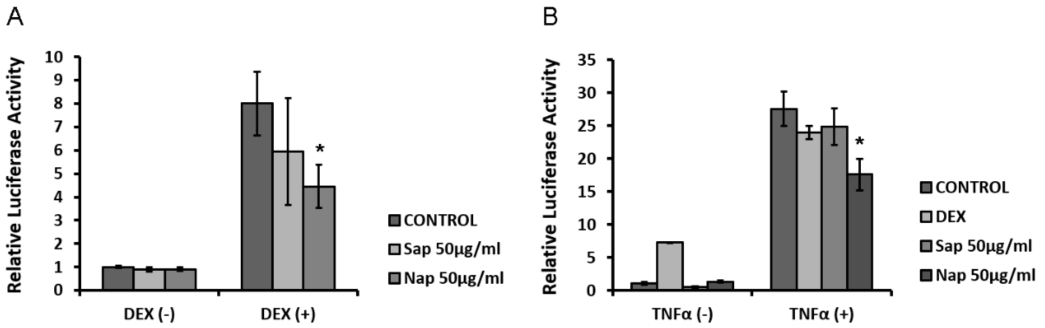

Figure S2: Assessment of the apolar fractions effect on (a) the DEX-induced GR transcriptional activation and (b) the TNFα-induced NF-κB transcriptional activation by the Southern and Northern fraction, at a concentration of 50μg/ml. Luciferase and β-galactosidase activity were measured in cell extracts from HEK293 in hormone-free medium, transiently co-transfected with a GRE-Luc or NF-κB-RE reporter gene construct and a β-galactosidase reporter construct and subsequently treated with 50μg/ml of the Southern and Northern apolar leaves fraction and/or 1μM DEX, for 6 hrs. Control cells were treated with DMSO (1:1000) and EtOH (1:1000). Relative luciferase activity was expressed as luciferase activity normalized against β-galactosidase activity. Data is expressed as mean ± SD, (n=3-6), * p<0.05; ** p<0.01, compared to relative controls.

1.3. mRNA isolation and real-time PCR

Cells were grown on 6 well plate, for 48 hours in hormone depleted medium and were further incubated for 4 hours in hormone depleted medium with 50μg/ml Mastiha tree polar and medium-polar leaves fractions and/or 0,1μM DEX. Subsequently, cells were washed with phosphate buffer saline and total RNA was extracted using Trizol followed by DNase treatment (Promega) and reverse transcription into cDNA, using random primers and superscript II reverse transcriptase (Invitrogen). Expressed levels of mRNA were quantified using real-time PCR and appropriate primers (Table S1), as previously described [3]. The SYBR Green qPCR super mix Universal (Invitrogen) products and the Step One Plus Real-Time PCR System was used for that purpose. Conditions for PCR were: 52°C for 2 min, 95°C for 2 min, 40 cycles of 95°C for 15 sec and 55°C for 20 sec, followed by 72°C for 20 sec according to manufacturer suggestions. Primers sequences for real-time PCR are shown in the table below:

	Forward primer	Reverse primer
GR	5′ CAGCTCCTCAACAGCAACAACA 3′	5′ GTGCTGTCCTTCCACTGCTC 3′
GAPDH	5′ CATGAGAAGTATGACAACAGCCT 3′	5′ AGTCCTTTCCACGATACCAAAGT 3′

Table S1: Primers used for real-time PCR measurement

To investigate the biochemical mechanism by which medium-polar- and polar leaves fractions from Chios *Pistacia Lentiscus* L. grown in the South or North Chios Greek island cause reduction in GR protein levels, fractions effect on GR mRNA levels was assessed. As shown in Figure 3, no statistically significant differences were observed on GR mRNA levels treated with 50μg/ml leaves extracts and/or 0,1μM DEX, for 4 hours.

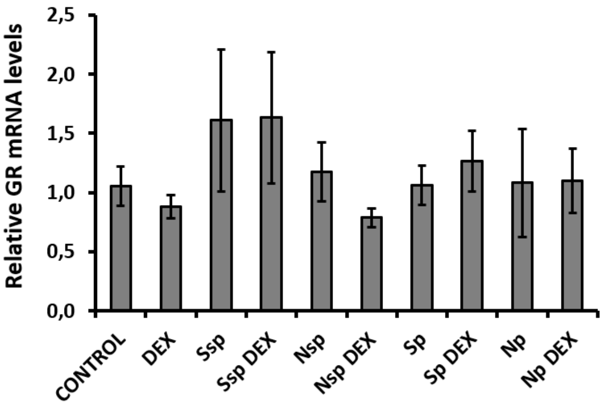

Figure S3: Real-time PCR was applied to evaluate mRNA levels of GR, in HEK293 cells, grown in hormone depleted medium, upon 4 hours treatment with 50µg/ml leaves medium-polar and polar fractions and/or 0,1µM DEX. GAPDH mRNA levels was also evaluated as reference conditions.

1.4 ^{1}H NMR analysis and characterization

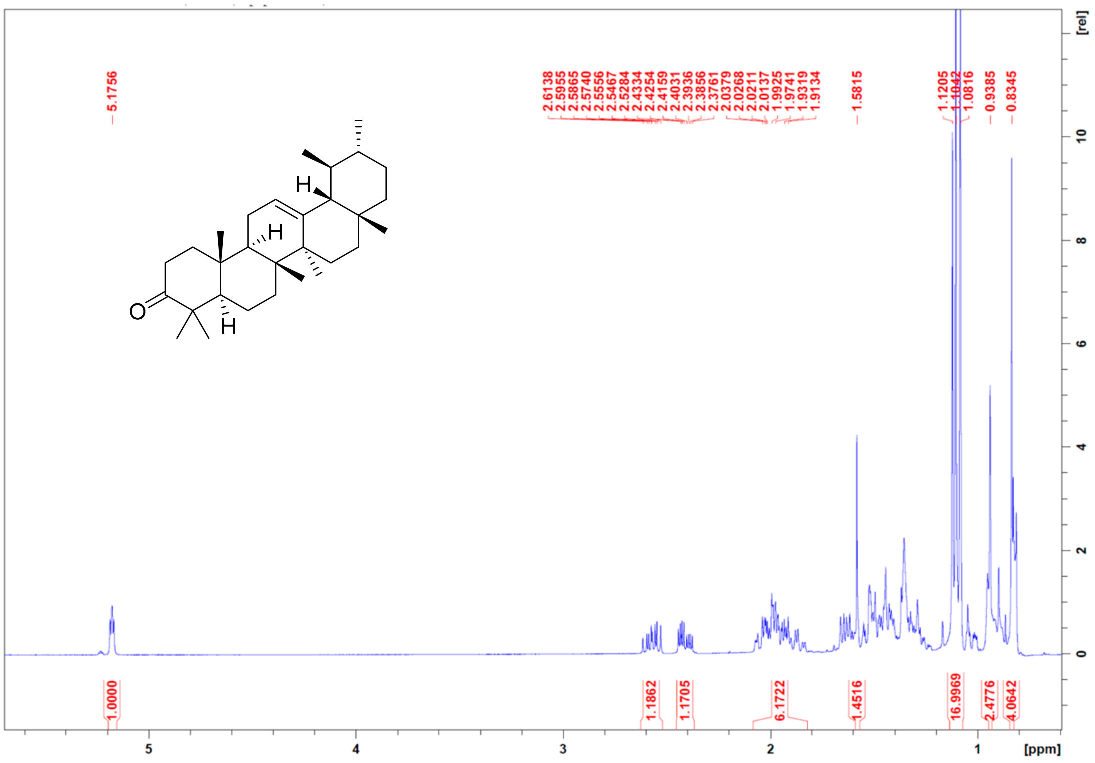

Figure S4: ^{1}H NMR of α-amyrenone in $CDCl_3$

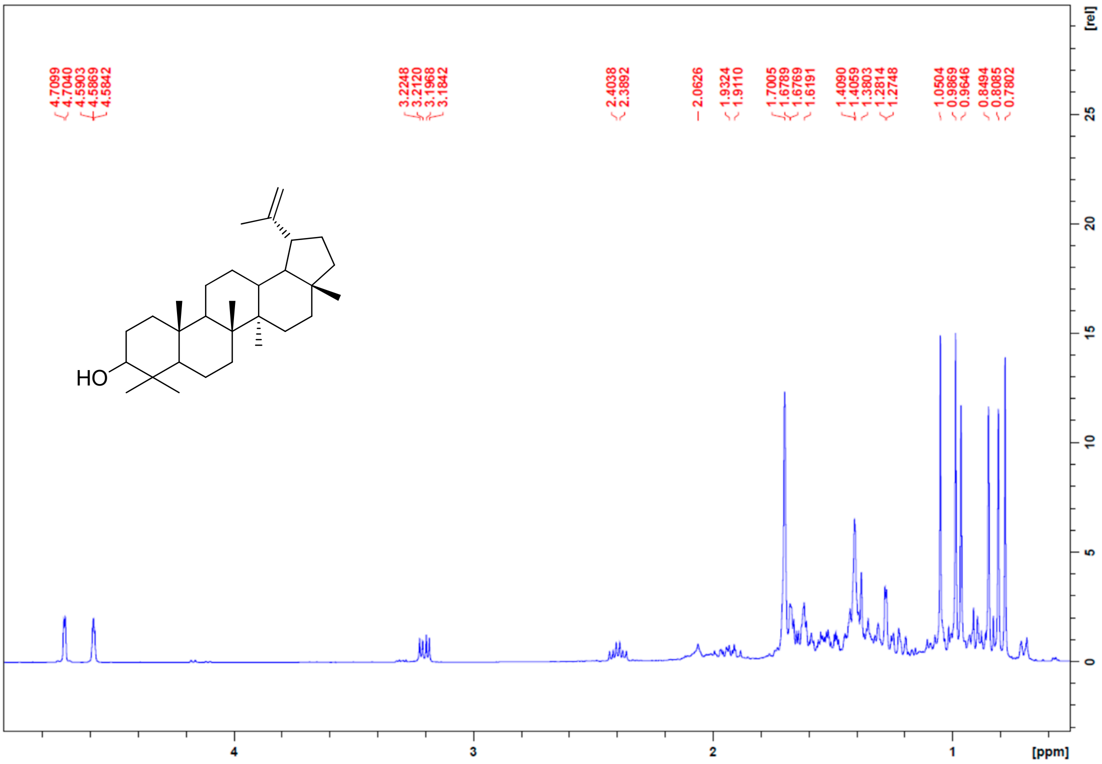

Figure S5. ^{1}H NMR of lupeol in $CDCl_3$

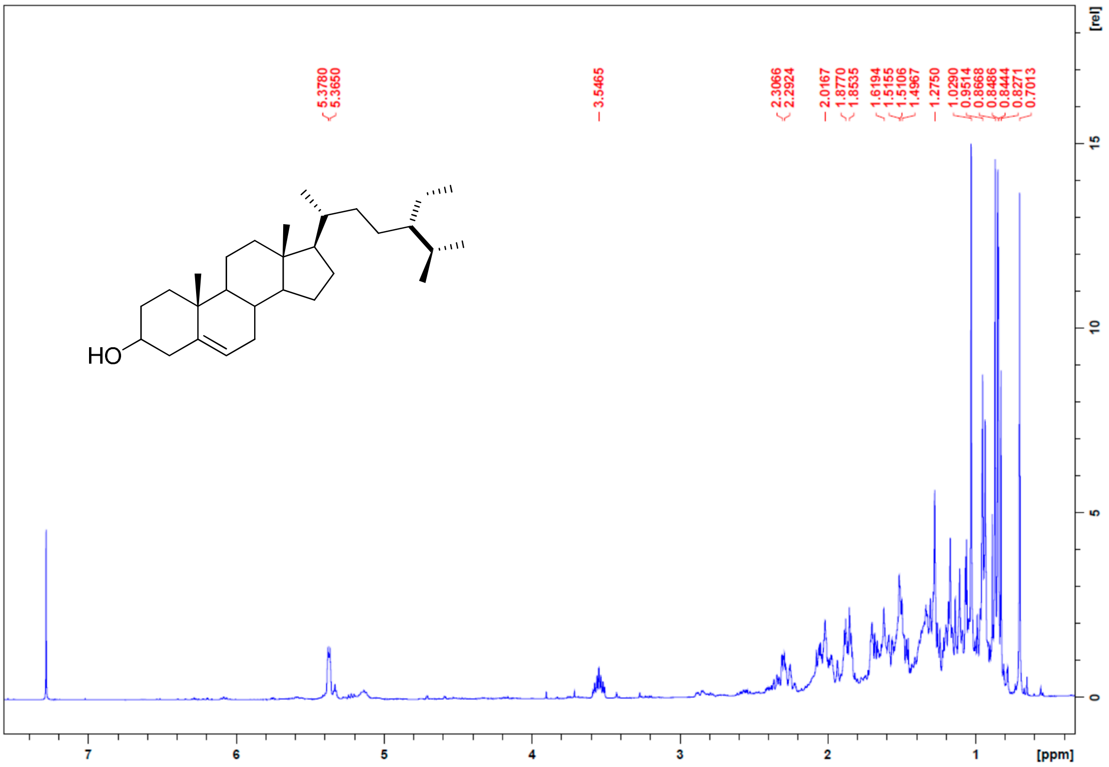

Figure S6: ^{1}H NMR of β-sitosterol in $CDCl_3$

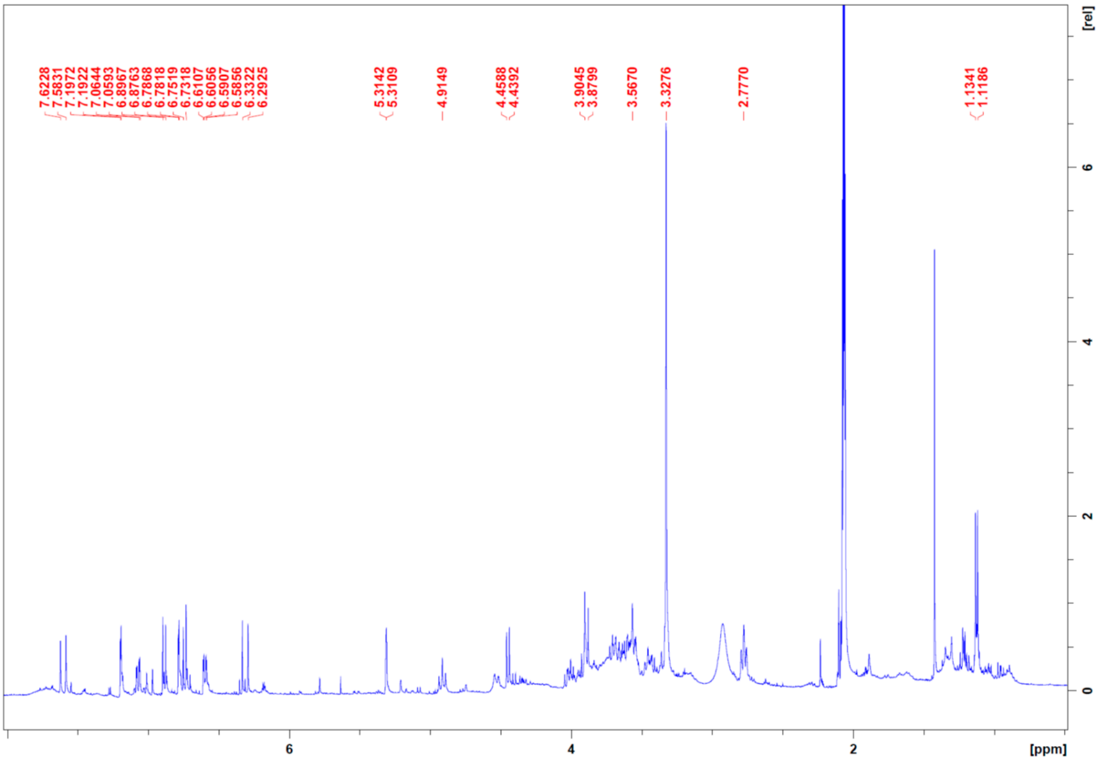

Figure S7. ^{1}H NMR of polar fraction in acetone$_{d6}$ from *P. lentiscus* leaves extract purified by RP C-18

References

1. Mosmann, T., Rapid colorimetric assay for cellular growth and survival: application to proliferation and cytotoxicity assays. *Journal of immunological methods* **1983,** 65, (1-2), 55-63. 10.1016/0022-1759(83)90303-4
2. Georgatza, D.; Gorgogietas, V. A.; Kylindri, P.; Charalambous, M. C.; Papadopoulou, K. K.; Hayes, J. M.; Psarra, A. G., The triterpene echinocystic acid and its 3-O-glucoside derivative are revealed as potent and selective glucocorticoid receptor agonists. *The international journal of biochemistry & cell biology* **2016,** 79, 277-287. 10.1016/j.biocel.2016.08.028
3. Gorgogietas, V. A.; Tsialtas, I.; Sotiriou, N.; Laschou, V. C.; Karra, A. G.; Leonidas, D. D.; Chrousos, G. P.; Protopapa, E.; Psarra, A. G., Potential interference of aluminum chlorohydrate with estrogen receptor signaling in breast cancer cells. *Journal of molecular biochemistry* **2018,** 7, (1), 1-13.
